# Antibacterial and Antibiotic Modifying Potential of Crude Extracts, Fractions, and Compounds from* Acacia polyacantha* Willd. against MDR Gram-Negative Bacteria

**DOI:** 10.1155/2019/7507549

**Published:** 2019-03-07

**Authors:** Flora T. Mambe, Jean Na-Iya, Ghislain W. Fotso, Fred Ashu, Bathélémy Ngameni, Bonaventure T. Ngadjui, Veronique P. Beng, Victor Kuete

**Affiliations:** ^1^Department of Biochemistry, Faculty of Science, University of Dschang, Dschang, Cameroon; ^2^Department of Biochemistry, Faculty of Science, University of Yaoundé I, Yaoundé, Cameroon; ^3^Ministry of Scientific Research and Innovation, Cameroon; ^4^Department of Organic Chemistry, Faculty of Science, University of Yaoundé I, Yaoundé, Cameroon; ^5^Department of Pharmacognosy and Pharmaceutical Chemistry, Faculty of Medicine and Biomedical Science, University of Yaoundé I, Yaoundé, Cameroon

## Abstract

The present study aimed to assess the* in vitro* antibacterial and antibiotic modifying activities of methanol extracts prepared from the leaf (APL) and bark (APB) of* Acacia polyacantha*, fractions (APLa-d) and compounds isolated from APL against a panel of multidrug resistant (MDR) Gram-negative bacteria. Leaf extract was subjected to column chromatography for compounds isolation; antibacterial assays were performed on samples alone and with an efflux pump inhibitor (EPI), respectively, and several antibiotics on the tested bacteria. The phytochemical investigation of APL led to the isolation of stigmasterol (**1**), *β*-amyrin (**2**), 3-*O-β*-_D_-glucopyranosylstigmasterol (**3**), 3-O-methyl-D-chiro-inositol (**4**), epicatechin (**5**), quercetin-3-*O*-glucoside (**6**), 3-*O*-[*β*-_D_-xylopyranosyl-(1→4)-*β*-_D_-galactopyranosyl]-oleanolic acid (**7**), and 3-*O*-[*β*-galactopyranosyl-(1→4)-*β*-_D_-galactopyranosyl]-oleanolic acid (**8**). APL and APB had minimal inhibitory concentration (MIC) values ≤ 1024 *μ*g/mL on 73.3% and 46.7% of the tested bacteria, respectively. APLb and APLd were effective against 88.9% of tested bacterial species with compound 8 showing the highest activity inhibiting 88.9% of tested bacteria. The EPI, phenylalanine-arginine-*β*-naphthylamide (PAßN), strongly improved the activity of APL, APLb, APLd, and compound** 8** on all tested bacteria. Synergistic effects were obtained when APL and compounds** 7** and** 8** were combined with erythromycin (ERY), gentamycin (GEN), ciprofloxacin (CIP), and norfloxacin (NOR). The present study demonstrates the antibacterial potential of* Acacia polyacantha* and its constituents to combat bacterial infections alone or in combination with EPI.

## 1. Introduction

Bacterial drug resistance constitutes a serious concern in the therapy of infectious diseases. Despite the abundance of various classes of antibiotics, the emergence of resistant strains of bacteria is increasing [1]. This phenomenon of resistance has increased the disease burden, and it has become necessary to search for new and cheaper alternatives with fewer side effects [2]. Botanicals (or crude plant extracts) and their secondary metabolites have long been used by humans for medicinal purposes. It is estimated that about 80% of the world's population uses medicinal plants as alternative for their health care [3]. Cameroon's flora is an enormous reservoir of antibacterial botanicals and phytochemicals (or plants secondary metabolites); some Cameroonian medicinal plants previously documented for their antibacterial potential include* Treculia obovoidea *[4],* Vismia laurentii* [5],* Artocarpus communis* [6],* Piper nigrum* and* Vernonia amygdalina* [7],* Cyperus esculentus* [8], and* Beilschmiedia obscura* [9]. Several botanicals have previously been reported for their activity against multidrug resistant (MDR) bacteria and had the ability to potentiate the activity of currently used antibiotics; such plants included* Dorstenia psilurus* [10],* Combretum molle* [11],* Xanthosoma mafaffa*,* Moringa oleifera,* and* Passiflora edulis* [12],* Rubus fellatae, *and* Manihot esculenta *[13]. It is important to improve our library of botanicals and phytochemicals with promising antibacterial potential, in order to combat MDR phenotypes. In the present study, we selected another Cameroonian medicinal plant,* Acacia polyacantha* Willd. (Fabaceae).* Acacia polyacantha* is a deciduous, straight cylindrical, erect tree of about 10-15 m height found in Tropical Africa. It has a geographical distribution, ranging from Gambia to Ethiopia and southwards to Kenya and Zimbabwe [14, 15]. The plant is traditionally used to treat livestock diseases and gastrointestinal infections [16]. The plant is also used as a remedy for snakebite and as an infusion to bath children who are restless at night [14]. This is the first report on the antibacterial potential of this plant against MDR bacteria. It was found that this plant had no anthelmintic effect against a levamisole resistant strain of the nematode* Caenorhabditis elegans* [17]. Previous phytochemical investigations of the leaves of the plant led to the isolation of polyacanthoside A, oleanolic acid, stigmasterol, stigmasterol-3-*O*-*β*-glucopyranosyl, epicatechin quercetin-3-*O*-glucoside, 3-O-methyl-D-Chiro-inositol, and 3-*O*-[*β*-_D_- galactopyranosyl-(1→4)-*β*-_D_-galactopyranosyl]-oleanolic acid [15]. The present study was designed to evaluate the antibacterial activity of the leaf and bark extracts of* Acacia polyacantha* against Gram-negative bacteria expressing MDR phenotypes. The work includes the isolation and identification of the active constituents of the leaf as well as the ability of this plant and its components to potentiate the activity of commonly used antibiotics.

## 2. Material and Methods

### 2.1. General Procedure

Optical rotation was measured with a Horiba SEPA-300 polarimeter (Horiba, Kyoto, Japan). NMR spectra were recorded on Bruker DMX Avance 600 instruments equipped with an autotune probe and using the automation mode aided by the Bruker program. HREI-SMS spectra were determined on a micrOTOF-Q 98 spectrometer (Bruker-Daltonics, Bilerica, MA). For column chromatography, silica gel 60 particles size 0.04–0.063 mm (Merck, Darmstadt, Germany) and Sephadex LH-20 purchased at Sigma-Aldrich (St Louis, MO) were used. The plates were visualized using UV (254 and 366 nm) and revealed by spraying with vanillin-sulphuric acid (1% ethanolic solution of vanillin + 10% ethanolic sulphuric acid).

### 2.2. Plant Material and Extraction

The bark and leaf of* Acacia polyacantha* Willd. (Fabaceae) were collected on February 2016 in Kaéle, in the Far North Region of Cameroon. The plant was then identified at the National Herbarium of Cameroon by Mr. Nana Victor and a voucher specimen was deposited under the registration number 58985/SRF/CAM. Air-dried and powdered leaf and bark of* A. polyacantha* (2 kg) were extracted twice at room temperature with 4 L of methanol (MeOH) for 48 hours. The solvent was evaporated under reduced pressure to yield 229 g and 108 g of crude leaf (APL) and bark (APB) extracts, respectively. These extracts were then kept at 4°C until further use.

### 2.3. Isolation of the Constituents from Leaves of* Acacia polyacantha*

Part of APL (225 g) was dissolved in a mixture of petroleum ether/ethyl acetate (99:1) and shaken to remove a dark green extract of chlorophyll. The residue (110 g) was subjected to silica gel column chromatography (40-63 *μ*m, 6 x 50 cm) using hexane-ethyl acetate (AcOEt) and chloroform (CHCl_3_)-MeOH gradients as eluents. 198 subfractions (frs) of 300 mL each were collected as follows: sub-frs 1-13 (hexane:AcOEt, 95:5), sub-frs 14-29 (hexane:AcOEt, 90: 10), sub-frs 30-63 (hexane:AcOEt,85:15), sub-frs 64-117 (hexane:AcOEt, 80:20), sub-frs 118-122 (hexane:AcOEt, 70:30), sub-frs 123-129 (hexane:AcOEt, 60:40), sub-frs 130-140 (CHCl_3_:MeOH, 97.5:2.5), sub-frs 141-152 (CHCl_3_:MeOH, 95:5), sub-frs 153-166 (CHCl_3_:MeOH, 90:10), sub-frs 167-182 (CHCl_3_:MeOH, 85:15), sub-frs 183-190 (CHCl_3_:MeOH, 80:20), and sub-frs 191-198 (CHCl_3_:MeOH, 75:25). These sub-frs were then pooled on the basis of their thin layer chromatography (TLC) profiles into four fractions as follows: APLa (sub-frs 1-34); APLb (sub-frs 35-171); APLc (sub-frs 172-183); and APLd (sub-frs 184-198). Upon antibacterial testing, fractions APLa, APLb, and APLd were selected for further purification.

Fraction APLa was column chromatographed over silica gel 60 column using increasing gradient of hexane:AcOEt, mixtures as eluents. 105 subfractions of 100 mL each were collected as follows: sub-frs 1-39 (hexane:AcOEt,95:5), sub-frs 40-87 (hexane:AcOEt,90:10), and sub-frs 88-105 (hexane:AcOEt,85:15). Compounds** 1** (45.9 mg) and** 2** (44.1 mg) were obtained as white powders after filtration from sub-frs 15-40 and sub-frs 41-90, respectively.

Fraction APLb was submitted to column chromatography (CC) over silica gel 60 using increasing gradient hexane:AcOEt, and CHCl_3_:MeOH, mixtures as eluents. 235 sub-fractions of 200 mL each were collected as follows: sub-frs 1-56 (hexane:AcOEt,85:15), sub-frs 57-133 (hexane:AcOEt,80:20), sub-frs 134-142 (hexane:AcOEt,70:30), sub-frs 143-155 (hexane:AcOEt,60:40), sub-frs 156-176 (CHCl_3_:MeOH, 97.5:2.5), sub-frs 177-199 (CHCl_3_:MeOH, 95:5), sub-frs 200-226 (CHCl_3_:MeOH, 90:10), and sub-frs 227-235 (CHCl_3_:MeOH, 85:15). Compound** 3** (83.2 mg) was obtained as white powder after filtration from sub-frs 1-30. Subfraction 31-235 were pooled together and were further purified over Sephadex LH-20 using isocratic CHCl_3_:MeOH, (7:3) as eluent. Sub-frs of 5 mL were collected. Sub-frs 6-11 afforded compound** 4** (6.11 mg) as a white powder while compound** 5** (18 mg) was isolated in sub-frs 35-50 as a red powder.

Fraction APLd was submitted to CC using silica gel 60 with increasing gradient of CHCl_3_:MeOH, mixtures as eluents. 45 sub-frs of 100 mL each were collected as follows: sub-frs 1-21 (CHCl_3_:MeOH, 80:20) and sub-frs 22-45 (CHCl_3_:MeOH, 75:25). Compound** 6** (30 mg) was obtained as yellow powder after filtration from sub-frs 1-13. Sub-frs 14-34 was further purified twice over Sephadex LH-20 using isocratic CHCl_3_:MeOH, (7:3) to afford compound** 7** (95.2 mg) as beige crystals. Sub-frs 35-42 was also purified similarly to sub-frs 14-34 for yield compound** 8** (10.2 mg) as a white powder.

### 2.4. Antibacterial Assays

#### 2.4.1. Chemicals for Antimicrobial Assay

Chemicals used included phytochemicals, reference antibiotics (RA), microbial growth indicator, and efflux pump inhibitor (EPI). Phytochemicals were stigmasterol, *β*-amyrin, stigmasterol-3-*O-β*-glucopyranosyl, 3-O-methyl-D-chiro-inositol, epicatechin, quercetin-3-*O*-glucoside, 3-*O*-[*β*-_D_-xylopyranosyl-(1→4)-*β*-_D_-galactopyranosyl]-oleanolic acid, and 3-*O*-[*β*-galactopyranosyl-(1→4)-*β*-_D_-galactopyranosyl]-oleanolic acid. They were isolated from the leaf of* Acacia polyacantha*. Their ^1^H and ^13^C nuclear magnetic resonance spectroscopy (NMR) spectra as well as their major chemical shifts are provided as supporting information (S1). The RA tested were chloramphenicol (CHL), ciprofloxacin (CIP), cloxacillin (CLX), doxyciclin (DOX), gentamycin (GEN), erythromycin (ERY), kanamycin (KAN), and norfloxacin (NOR) obtained from Sigma-Aldrich, St. Quentin Fallavier, France. The microbial growth indicator used was iodonitrotetrazolium ≥ 97% (INT, Sigma-Aldrich) while the EPI was phenylalanyl-arginine-*β*-naphthylamide (PAßN) (Sigma-Aldrich). Dimethyl sulfoxide (Sigma-Aldrich) was used to dissolve chemicals.

#### 2.4.2. Microbial Strains and Culture Media

In this study, 15 Gram-negative bacterial strains belonging to five species were used. They included reference (from American Type Culture Collection, ATCC) and clinical (Laboratory collection) strains of* Escherichia coli *(ATCC8739, ATCC10536, AG102, and AG100Atet),* Enterobacter aerogenes *(ATCC13048, CM64, EA27 and EA289),* Klebsiella pneumoniae *(ATCC11296, KP55 and KP63),* Providencia stuartii* (ATCC29916 and NEA16), and* Pseudomonas aeruginosa *(PA01 and PA124). Bacterial features or resistance profiles previously reported [19] are shown as supporting information (Table S2). Bacterial cultures were maintained on agar plates at 4°C and subcultured on a fresh appropriate agar plates 24 h prior to any antimicrobial assay. The activation of bacteria prior to any assay was done in Mueller Hinton Agar (Sigma) meanwhile antibacterial assays were carried out using Mueller Hinton broth (MHB; Sigma) [20].

#### 2.4.3. Determination of Minimum Inhibitory Concentration (MIC) and Minimum Bactericidal Concentration (MBC)

The MICs and MBCs of extract, fractions and isolated compounds against the tested bacteria were determined by microplate dilution method using the rapid INT colorimetric assay according to previously described methods [21] with some modifications [19, 22, 23]. In general, the concentrations ranges were 8-1024 *μ*g/mL for crude extracts, 4-512 *μ*g/mL for fractions, and 2-256 *μ*g/mL for chloramphenicol.

The role of efflux pumps in the susceptibility of Gram-negative bacteria to the most active samples (APL, APLb, APLd, compound** 8** and CHL) was evaluated by testing the studied samples in the presence of an EPI, PA*β*N (at 30 *μ*g/mL) using the rapid INT colorimetric assay as earlier described [7, 19]. A preliminary study showed that the concentration of 30 *μ*g/mL did not affect the growth of selected bacteria [23]. Nine selected bacterial strains including* E. coli *ATCC8739 and AG102,* E. aerogenes *ATCC13048 and CM64,* K. pneumoniae *KP55 and KP63,* P. aeruginosa *PA01 and PA124 and* P. stuartii *ATCC29916 were used. Increase of activity was determined as the ratio of MIC in the absence of EPI versus MIC in the presence of EPI.

To evaluate the potentiating or antibiotic resistance modulating effect of samples, a preliminary assay was performed against a problematic bacterium,* P. aeruginosa* PA124 (see supporting information S3); the selected samples were tested at various subinhibitory concentrations in combination with antibiotics. MIC/2 and MIC/4 were selected as the best subinhibitory concentrations [6, 24] and were further used for the best samples (APL, compounds** 7** and** 8**) in combination with antibiotics against the seven other bacteria. Briefly, the MIC was determined as described above. The 96-wells microplate rows receiving antibiotic dilutions without extracts were used for the determination of the MICs of the antibiotics. The concentrations ranges of antibiotics were generally 2-256 *μ*g/mL. The MIC was determined as described using INT colorimetric method as earlier described [3, 19]. The modulation factor was defined as the ratio of the MIC for the antibiotic alone and that of the antibiotics in the presence of the extract (RHL). Modulation factor ≥ 2 was set as the cutoff for biological significance of antibiotic resistance modulating effects [19, 20].

## 3. Results

### 3.1. Phytochemistry

**Figure 1:**
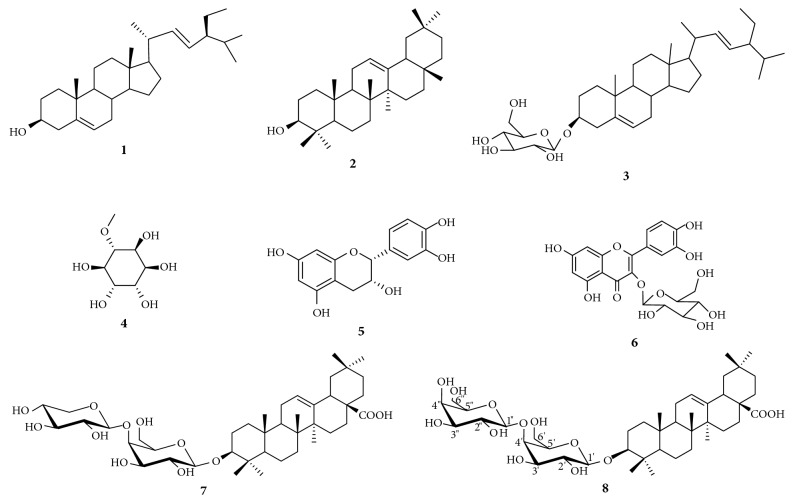
Chemical structures of compounds isolated from the leaf of* Acacia polyacantha*.** 1:** stigmasterol,** 2:***β*-amyrin,** 3:** 3-*O-β*-_D_-glucopyranosylstigmasterol,** 4:** 3-O-methyl-D-chiro-inositol,** 5:** epicatechin,** 6:** quercetin-3-*O*-glucoside,** 7:** 3-*O*-[*β*-_*D*_-xylopyranosyl-(1→4)-*β*-_D_-galactopyranosyl]-oleanolic acid, and** 8:** 3-*O*-[*β*-galactopyranosyl-(1→4)-*β*-_D_-galactopyranosyl]-oleanolic acid.

The chemical structures of compounds isolated from the leaf of* Acacia polyacantha* were determined using NMR (^1^H and ^13^C) data, in comparison with the literature (Figure 1). Compounds were identified as stigmasterol C_29_H_50_O (**1;** melting point (m.p.): 134-135°C; m/z 414) [21], *β*-amyrin C_30_H_50_O (**2;** m.p.: 187-190°C; m/z 426) [25], 3-*O-β*-_D_-glucopyranosylstigmasterol C_35_H_58_O_6_ (**3;** m.p.: 272-274°C; m/z 412) [26], 3-O-methyl-D-chiro-inositol C_7_H_14_O_6_ (**4;** m.p.: 181°C;* m/z* 217; [*α*]_D_^25^: +60,00) [24], epicatechin C_15_H_14_O_6_ (**5;** m.p.: 345-350°C;* m/z* 270) [27], quercetin-3-*O*-glucoside C_21_H_20_O_12_ (**6;** m.p.: 230-232°C;* m/z* 464) [28], 3-*O*-[*β*-_D_-xylopyranosyl-(1→4)-*β*-_D_-galactopyranosyl]-oleanolic acid C_41_H_66_O_12_ (**7;** m.p.: 216-217°C;* m/z* 773; [*α*]_D_^25^: +23,2° (c 1,25; MeOH)) [15] and 3-*O*-[*β*-galactopyranosyl-(1→4)-*β*-_D_-galactopyranosyl]-oleanolic acid C_42_H_68_O_13_ (**8;** amorphous powder;* m/z* 803) [21]. The ^1^H NMR, ^13^C NMR spectra and major chemical shifts of these compounds are available as supplementary data (S2).

### 3.2. Antibacterial Activity

Extracts, fractions, and isolated compounds were tested for their antimicrobial activity against the studied Gram-negative bacteria. The results are shown in Tables 1 and 2. APL and APB had MIC values ≤ 1024 *μ*g/mL on 11/15 (73.3%) and 7/15 (46.7%) tested bacteria. MIC values of CHL varied between 2-256 *μ*g/mL (Table 1). APLc as well as compounds** 1**,** 2** and** 3** were not active (Table 2). MIC values ≤ 512 *μ*g/mL for fractions and ≤256 *μ*g/mL for compounds were obtained against 8/9 (88.9%) tested bacteria for both APLb and APLd and against 7/9 (77.8%) for compound** 8** (Table 2). Analysis of data from Tables 1 and 2 indicated bacteriostatic effects as MBC/MIC ratios were generally above 4 with no MBC value ≤ 1024 *μ*g/mL recorded for the crude extract, fractions, and compounds.

### 3.3. Role of Efflux Pumps in the Susceptibility of Gram-Negative Bacteria Botanicals and Phytochemicals

The most active extracts, fractions, isolated compound and RA (APL, APLb, APLd, Compound** 8**, CHL) were tested in the presence of EPI against 9 bacterial strains including reference strains and MDR phenotypes (Table 3). The results showed that PA*β*N improves the activity (decrease of MIC values) of APL, APLb, APLd and compound** 8** on all tested bacteria with the highest MIC values of 256 *μ*g/mL for crude extract (APL) and compound** 8**, and 64 *μ*g/mL for fractions (Table 3). A preliminary study showed that the MIC of PA*β*N was above 256 *μ*g/mL on the selected bacteria and that the concentration of 30 *μ*g/mL did not affect their growth.

### 3.4. Antibiotic Resistance Modulating Effects of Botanicals and Phytochemicals

A preliminary study against* P. aeruginosa* PA124 (see supporting information S3) allowed choosing the appropriate subinhibitory concentrations of MIC/2 and MIC/4 as well as APL, compounds** 7** and** 8** for further studies. These samples were combined with eight antibiotics to evaluate their possible synergistic effects. The results summarized in Tables 4, 5, and 6 showed that the synergistic effects were noted with all the tested samples and many antibiotics. When tested at their MIC/2, the percentages of bacterial strains on which synergism was observed (PBS) were ≥50% when APL was combined with ERY and CIP (Table 4), when compound** 7** was combined with ERY, KAN and GEN (Table 5), and when compound** 8** was combined with ERY, and DOX (Table 6). At their MIC/4, the PBS ≥50% was obtained when APL was combined with GEN and CIP (Table 4), when compound** 7** was combined with ERY and GEN (Table 5), and when compound** 8** was combined with ERY and NOR (Table 6).

## 4. Discussion

### 4.1. Phytochemistry

The isolated compounds included five terpenoids amongst which were one sterol (stigmasterol;** 1**), one triterpene (*β*-amyrin;** 2**), and three saponins [stigmasterol-3-*O-β*-glucopyranosyl (**3**),** 7:** 3-*O*-[*β*-_D_-xylopyranosyl-(1→4)-*β*-_D_-galactopyranosyl]-oleanolic acid, (**7**) and* O*-[*β*-galactopyranosyl-(1→4)-*β*-_D_-galactopyranosyl]-oleanolic acid (**8**)], two flavonoids: epicatechin (**5**) and quercetin-3-*O*-glucoside (**6**) and one sugar, 3-O-methyl-D-chiro-inositol (**4**). Previous phytochemical investigations of the leaf of the plant led to the isolation of polyacanthoside A, oleanolic acid, epicatechin, quercetin-3-*O*-glucoside as well as compounds** 1**,** 4, 3, 5** and** 6** and** 8** [15]. However, few compounds were reported in this study, probably due to the fact that we focussed only on the bioactive fractions of the leaf extract; meanwhile, less active fractions were not further investigated.

### 4.2. Antibacterial Activity

It is important to take into consideration the development of resistance by Gram-negative bacteria when searching for new antimicrobial agents. In the present study, several clinical MDR bacteria expressing MDR phenotypes were used. The MIC values of chloramphenicol were above 10 *μ*g/mL on most of the bacterial strains (Table 1), confirming their resistance phenotypes. Established cutoff points for antibacterial activity of botanicals consider that the inhibitory effect is significant when MIC values are below 100 *μ*g/mL, moderate when 100 ≤ MIC ≤ 625 *μ*g/mL, and weak when MIC > 625 *μ*g/mL [18, 29]. On this basis, the antibacterial activity of the crude extracts (APL and APB) could mostly be considered as moderate or poor (Table 1). Nevertheless, MIC values below 100 *μ*g/mL were obtained with APL on the problematic bacterial strain* P. aeruginosa* (PA01) and* P. stuartii *NAE16 (Table 1), as well as with APB against* E. aerogenes *ATCC13048. This data suggested that these extracts could be useful to fight bacterial infections, especially in traditional medicine where they are utilised. It is worth nothing that the MIC values of APL against* P. aeruginosa* (PA01) and* P. stuartii* ATCC29916, or APB against* E. aerogenes* ATCC13048 (Table 1) were lower than those of chloramphenicol, confirming this hypothesis. This was the rational for carrying out bioguided fractionation in order to isolate more active compounds from the leaf extract. Fractions APLa, APLb, and APLd had MIC values below 100 *μ*g/mL against 1, 3, and 7 of the 9 tested bacterial species, respectively (Table 2). This was an indication that fractionation led to more active samples. The activity of phytochemicals was set as significant when MIC<10 *μ*g/mL, moderate when 10<MIC<100 *μ*g/mL, and low when MIC>100 *μ*g/mL [18, 29]. However, less active compounds were obtained from the purified fractions, with none of them displaying MIC value below 10 *μ*g/mL (Table 2). This was an indication that constituents of the extract and fractions synergistically inhibited bacterial growth. All tested samples had MBC/MIC ratios above 4 (Tables 1 and 2), showing that they mostly exerted bacteriostatic effects [30]. To the best of our knowledge, the antibacterial activity of* A. polycantha *as well as that of its most active constituent (compound** 8**) was reported for the first time.

Concerning the structure-activity relationship, it appeared that terpenoids** 1** and** 2** (with no sugar in their chemical structures) and** 3** (with only one sugar) as well as the polyol (**4**) were devoid of antibacterial activity (Table 2). Flavonoids** 5** and** 6** as well as saponins** 7** and** 8** had selective and poor antibacterial. Within saponins, it can be noted that the presence of a second galactopyranosyl substituent (compound** 8**) instead of xylopyranosyl (compound 7) significantly increased the antibacterial activity, with compound** 8** displaying MIC values ≤ 64 *μ*g/mL against 7/9 tested bacteria versus 0/9 for compound** 7** (Table 2).

### 4.3. Role of Bacterial Efflux Pumps

The clinical MDR bacteria tested in this work overexpressed efflux mechanism* via* the efflux pumps of the resistance nodulation cell division (RND) family, namely, AcrAB-TolC for enterobacteria such as* E. coli, E. aerogenes, K. pneumoniae, *and* P. stuartii *and MexAB-OprM for* P. aeruginosa* [31–36]. These efflux pumps expel toxic compounds (including antibiotics) out of the bacterial cytoplasm, preventing them from reaching their intracellular target [37]. Efflux Pump Inhibitors, such as Pa*β*N, could be used to restore the intracellular concentration of antibacterials acting on intracellular target by blocking the bacterial efflux pumps. In the presence of PA*β*N, it was observed that the activity of the crude extract (except against* P. aeruginosa *PA01), fractions APLb and APLd as well as compound** 8** and CHL strongly increased on almost all tested bacteria (Table 3). The fold increase ranged from 2 to ≥ 4 for APLb, from ≥ 1 to 8 for CHL, from 2 to ≥ 128 for compound** 8**, from 0.5 to 64 for APL, from 2 to 128 for APLd. This clearly indicated that compound** 8** as well as other active constituents of the APL are substrates of bacterial efflux pumps and that they may have an intracellular target [38]. Consequently, the development of an antibacterial drug combination of compound** 8**, as well as extracts or fractions with an EPI, could be an interesting strategy to tackle MDR bacterial infections. In effect, modulation factor ≥ 2 define a biologically significant antibiotic resistance modulating substance [19, 20]. Previous study demonstrated that PA*β*N could also restore the activity of several natural compounds on MDR bacteria expressing active efflux pumps, with MIC values decreasing below 10 *μ*g/mL in most of the tested bacteria for the coumarin, MAB3, the xanthone, laurentixanthone B, the naphthoquinones: diospyrone and plumbagin and the flavonoids: 4-hydroxylonchocarpin and isobavachalcone [22, 23].

### 4.4. Antibiotic Resistance Modulating Effects

Difficulties in the field of novel antibacterial drug discovery, for combating resistant pathogens, have propelled the search for new alternative medicine to improve or to restore the activity of commonly used antibiotics. Combining antibiotics with botanicals and phytochemicals is an attractive strategy as regards the diversity of secondary metabolites from natural source. If an antibacterial substance improves the activity of at least 70% of the tested antibiotics on more than 70% tested bacterial strains, it might be considered as a potential efflux pump inhibitor [39]. However, this was not the case in the present study, as neither APL nor compounds** 7** and** 8** were able to exert such degree of synergistic effects with antibiotics (Tables 4–6). However, synergistic effects were observed between APL, compounds** 7** and** 8** with at least one of the eight tested antibiotics against at least 50% of the MDR bacterial strains (Tables 3–6). This suggests that possible combination of these samples with specific antibiotics could help in antibacterial chemotherapy.

## 5. Limitations

Our study has limitations. The toxicity of this plant also needs to be performed to evaluate its safety.

## 6. Conclusion

In the present study, the antibacterial activity of the crude extract, fractions, and compounds from the leaf of* Acacia polyacantha* Willd. (Fabaceae) was investigated. It was found that the leaf extract was more active than the bark extract. The antibacterial constituents of the leaf extract include epicatechin (**5**), quercetin-3-*O*-glucoside (**6**), 3-*O*-[*β*-_D_-xylopyranosyl-(1→4)-*β*-_D_-galactopyranosyl]-oleanolic acid (**7**), and 3-*O*-[*β*-galactopyranosyl-(1→4)-*β*-_D_-galactopyranosyl]-oleanolic acid. Saponin** 8** was the major antibacterial constituent of the plant and acted as a substrate of bacterial EPI. Although the crude extract and its constituents were not EPI, they showed synergistic effects with several antibiotics and could be selectively used in bacterial chemotherapy. The overall results demonstrated that* Acacia polyacantha* is a source of antibacterial drug that should be explored further to develop novel substances to combat both sensitive and MDR phenotypes.

## Figures and Tables

**Table 1:** Minimal inhibitory concentration (MIC) and minimal bactericidal concentration (MBC) in *μ*g/mL of crude leaf and bark extracts, isolated compounds and chloramphenicol against reference strains and MDR Gram-negative bacteria.

Bacterial strains	Tested samples, MIC and MBC (in bracket) values (*µ*g/mL)		
	Leaf extract (APL)	Bark extract (APB)	Chloramphenicol
*Escherichia coli*			
ATCC8739	1024 (-)	512 (-)	**2** (64)
AG102	512 (-)	-	32 (256)
AG100Atet	512 (-)	-	32 (256)
ATCC10536	1024 (-)	-	**2** (32)
*Enterobacter aerogenes*			
ATCC13048	256 (-)	**8** (-)	16 (128)
CM64	-	-	256 (-)
EA27	1024 (-)	256 (-)	32 (256)
EA289	-	-	32 (256)
*Klebsiella pneumoniae*			
ATCC11296	1024 (-)	-	32 (256)
KP55	-	128 (-)	64 (256)
KP63	-	-	32 (256)
*Pseudomonas aeruginosa*			
PA01	**64** (-)	256 (-)	64 (-)
PA124	256 (-)	512 (-)	256 (-)
*Providencia stuartii*			
NEA16	1024 (-)	-	64 (256)
ATCC29916	**8** (-)	256 (-)	64 (256)

(-): MIC or MBC value > 1024 *µ*g/mL for APL and APB or >256 for chloramphenicol; MIC values in bold: significant antibacterial effect [18].

**Table 2:** MICs and MBCs in *µ*g/mL of fractions and compounds isolated from the leaf (APL) of *Acacia polyacantha* and chloramphenicol against a panel of selected Gram-negative bacteria.

Bacterial strains	Tested samples MIC and MBC (in bracket) values (*µ*g/mL)								
	Fractions from APL			Isolated compounds					Reference antibiotic
	APLa	APLb	APLd	**4**	**5**	**6**	**7**	**8**	chloramphenicol
*Escherichia coli*									
ATCC8739	-	**16** (-)	**16** (-)	-	-	-	-	32 (-)	**2** (64)
AG102	-	128 (-)	**64** (-)	256 (-)	-	128 (-)	-	32 (-)	32 (256)
*Enterobacter aerogenes*									
ATCC13048	-	256 (-)	**8** (-)	128 (-)	64 (-)	-	-	32 (-)	16 (128)
EA27	-	-	128 (-)	256 (-)	256 (-)	-	-	-	32 (256)
*Klebsiella pneumoniae*									
ATCC11229	**16** (-)	**64** (-)	**16** (-)	64 (-)	32 (-)	-	-	32 (-)	32 (256)
KP55	-	**16** (-)	**64** (-)	128 (-)	256 (-)	-	128 (-)	32 (-)	64 (256)
*Pseudomonas aeruginosa*									
PA01	-	128 (-)	**64** (-)	-	-	-	-	-	64 (-)
PA124	-	256 (-)	**64** (-)	256 (-)	128 (-)	-	-	32 (-)	256 (-)
*Providencia stuartii*									
ATCC29916	-	128 (-)	-	-	-	-	-	64 (-)	64 (256)

(-): > 512 *µ*g/ml for fractions or > 256 *µ*g/ml for compounds; APL: leaf extract; APLa, APLb, and APLd are fractions from APL; **1:** stigmasterol, **2:**  *β*-amyrin, **3:** 3-*O-β*-_D_-glucopyranosylstigmasterol, **4:** 3-O-methyl-D-chiro-inositol, **5:** epicatechin, **6:** quercetin-3-*O*-glucoside, **7:** 3-*O*-[*β*-_D_-xylopyranosyl-(1→4)-*β*-_D_-galactopyranosyl]-oleanolic acid, **8:** 3-*O*-[*β*-galactopyranosyl-(1→4)-*β*-_D_-galactopyranosyl]-oleanolic acid; MIC values in bold: significant antibacterial effect [18]. Notes: MICs of fraction APLc > 512 *µ*g/ml and MIC of compounds **1, 2, 3** were > 256 *µ*g/ml in all tested bacteria.

**Table 3:** MICs (in *μ*g/mL) of crude leaf extract (APL), active fractions (APLa and APLd), 3-*O*-[*β*-galactopyranosyl-(1→4)-*β*-_D_-galactopyranosyl]-oleanolic acid (**8**) and chloramphenicol in the presence of PAßN and fold increase of activity (in parenthesis) against a panel of selected Gram-negative bacteria.

Bacterial strains	Tested samples, MIC in *μ*g/mL in presence of PAßN and fold increase of activity (in parenthesis)				
	Leaf extract	Fractions from APL		Isolated compound	Reference antibiotic
	APL	APLb	APLd	**8**	Chloramphenicol
*Escherichia coli*					
ATCC8739	256 **(4)**	**4 (4)**	**2 (8)**	**≤2 (≥ 16)**	**≤ 2 (≥ 1)**
AG102	**64 (8)**	**32 (2)**	**8 (16)**	**≤ 2 (≥ 16)**	**4 (8)**
*Enterobacter aerogenes*					
ATCC13048	**64 (4)**	**4 (2)**	**≤4 (≥ 64)**	**≤ 2 (≥ 16)**	**2 (8)**
EA27	16** (64)**	**64 (2)**	**4 (128)**	64 **(4)**	**4 (8)**
*Klebsiella pneumoniae*					
ATCC11229	128 **(8)**	**≤ 4 (≥ 4)**	**≤ 4 (≥ 16)**	**≤ 2 (≥ 16)**	**4 (8)**
KP55	128 **(8)**	**32 (2)**	**4 (4**)	**≤ 2 (≥ 16)**	16 **(4)**
*Pseudomonas aeruginosa*					
PA01	128 (0.5)	**32 (2)**	**32(4)**	**≤2 (≥ 128)**	**8 (8)**
PA124	128 **(2)**	**32 (2)**	128 **(2)**	16 **(2)**	64 **(4)**
*Providencia stuartii*					
ATCC29916	**≤ 4 (**≥** 2)**	**32 (16)**	**32(4)**	16 **(4)**	16 **(4)**

(-): MIC > 1024 *μ*g/mL for crude extract APL, >512 for fractions APLb and APLd, 256 *μ*g/mL for 3-*O*-[*β*-galactopyranosyl-(1→4)-*β*-_D_-galactopyranosyl]-oleanolic acid (**8**); (): values in parenthesis are fold increase of activity in the presence of PAßN at 30 *μ*g/mL; MIC values in bold: significant antibacterial effects for MIC [18] and significant increase of activity in the presence of PAßN [19, 20]; fold increase of activity determined MIC of sample tested alone (Tables 1 and 2) versus MIC of the sample in the presence of PAßN. The MIC of PAßN was >256 mg/L for *E. coli*, *E. aerogenes*, *K. pneumoniae*, *P. aerogenes,* and *P. stuartii* strains.

**Table 4:** Antibiotic resistance modulatory activity of leaf extract (APL) of *Acacia polyacantha* against MDR Gram-negative bacteria.

Antibiotics	Extract concentration		Bacteria, MIC (*μ*g/mL) and modulating factors of the leaf extract (in bracket)							
		ATCC8739	AG102	ATCC13048	CM64	KP55	KP63	PA124	NEA16	PBS (%)
ERY	0	128	256	256	256	256	256	256	256	
	MIC/2	256 (0.5)^a^	**128 (2)** ^S^	**128 (2)** ^S^	256 (1)^I^	256 (1)^I^	**32 (8)** ^S^	> 256 (> 1)^a^	**128 (2)** ^S^	50
	MIC/4	256 (0.5)^a^	256 (1)^I^	256 (1)^I^	**128 (2)** ^S^	256 (1)^I^	**128 (2)** ^S^	256 (1)^I^	**128 (2)** ^S^	37.5

CLX	0	256	1024	> 1024	> 1024	1024	512	> 1024	> 1024	
	MIC/2	> 1024 (> 0.25)^a^	> 1024 (> 1)^a^	> 1024 (≥ 1)^I^	> 1024 (≥ 1)^I^	> 1024 (>1)^a^	> 1024 (> 0.5)^a^	> 1024 (≥ 1)^I^	> 1024 (≥ 1)^I^	0
	MIC/4	> 1024 (> 0.25)^a^	> 1024 (> 1)^a^	> 1024 (≥ 1)^I^	> 1024 (≥ 1)^I^	> 1024 (> 1)^a^	> 1024 (> 0.5)^a^	> 1024 (≥ 1)^I^	**1024 (1)** ^S^	12.5

KAN	0	16	16	< 2	16	4	64	8	64	
	MIC/2	128(0.125)^a^	64 (0.25)^a^	4 (0.5)^a^	16 (1)^I^	**< 2 (2)** ^S^	128 (0.5)^a^	8(1)^I^	**< 2( 32)** ^S^	25
	MIC/4	128(0.125)^a^	32 (0.5)^a^	8 (0.25)^a^	**8 (2)** ^S^	**< 2 (2)** ^S^	128 (0.5)^a^	8(1)^I^	**16 (4)** ^S^	37.5

GEN	0	32	16	16	16	256	32	128	256	
	MIC/2	> 256 (> 0.125)^a^	> 256 (> 0.062)^a^	16 (1)^I^	16 (1)^I^	256 (1)^I^	> 256 (> 0.125)^a^	**64 (2)** ^s^	**16 (16)** ^S^	25
	MIC/4	> 256 (> 0.125)^a^	**< 2(8)** ^S^	16 (1)^I^	16 (1)^I^	**< 2 (128)** ^S^	256 (1)^I^	**64 (2)** ^s^	**16 (16)** ^S^	50

DOX	0	< 1	4	16	16	4	8	64	32	
	MIC/2	2 (0.5) ^a^	8 (0.5) ^a^	32 (0.5)^a^	16 (1)^I^	4 (1)	**4 (2)** ^S^	**32 (2)** ^S^	> 128 (> 025)^a^	25
	MIC/4	< 1 (≤ 1)^I^	8 (0.5)^a^	32 (0.5)^a^	16 (1)^I^	**< 1 (> 4)** ^S^	8 (1)^I^	64 (1)^I^	> 128 (> 025) ^a^	14.28

CHL	0	2	32	16	256	64	32	256	64	
	MIC/2	2 (1)^I^	32 (1)^I^	> 16 (> 1)^a^	256 (1)^I^	64 (1)^I^	32 (1)^I^	256 (1)^I^	64 (1)^I^	0
	MIC/4	2 (1)^I^	32 (1)^I^	> 16 (> 1)^a^	256 (1)^I^	6 (1)^I^	32 (1)^I^	256 (1)^I^	64 (1)^I^	0

NOR	0	4	8	32	32	8	8	64	32	
	MIC/2	32 (0.125)^a^	64 (0.125)^a^	**16 (2)** ^S^	32 (1)^I^	64 (0.125)^a^	64 (0.125)^a^	**32 (2)** ^S^	32 (1)^I^	25
	MIC/4	> 128 (> 0.031)^a^	> 128(> 0.062)^a^	**16 **(2)^S^	32 (1)^I^	> 128 (> 0.062)^a^	> 128 (> 0.062)^a^	**32 (2)** ^S^	32 (1)^I^	25

CIP	0	< 0.5	1	8	8	< 0.5	1	64	8	
	MIC/2	16 (0.031)^a^	16 (0.0625)^a^	**4 (2)** ^S^	**4 (2)** ^S^	16 (0.031)^a^	8 (0.125) ^a^	**16 (4)** ^S^	**4 (2)** ^S^	50
	MIC/4	16 (0.031)^a^	16 (0.0625)^a^	**4 (2)** ^S^	**4 (2)** ^S^	16 (0.031)^a^	8 (0.125)^a^	**16 (4)** ^S^	**4 (2)** ^S^	50

ERY: erythromycin; CLX: cloxacillin; KAN: kanamycin; GEN: gentamycin; DOX: doxyciclin; CHL: chloramphenicol; NOR: norfloxacin; CIP: ciprofloxacin; bacterial strains: *Escherichia coli* (AG102, ATCC8739); *Enterobacter aerogenes* (ATCC13048, CM64); *Klebsiella pneumoniae* (KP55, KP63); *P. aeruginosa *PA124; *Providencia stuartii* NEA16; PBS: percentage of bacteria strain on which synergism has been observed. (): ameliorating factor of the antibiotics after association with APF; S: synergy; I: indifference; a: antagonist; values in bold: case of synergy [19, 20].

**Table 5:** Antibiotic resistance modulatory activity of 3-*O*-[*β*-_D_-xylopyranosyl-(1→4)-*β*-_D_-galactopyranosyl]-oleanolic acid (**7**) against MDR Gram-negative bacteria.

Antibiotics	Extract concentration	Bacteria, MIC (*μ*g/mL) and modulating factors of compound **7** (in bracket)								
		ATCC8739	AG102	ATCC13048	CM64	KP55	KP63	PA124	NEA16	PBS (%)
ERY	0	128	256	256	256	256	256	256	256	
	MIC/2	256 (0.5)^a^	**128 (2)** ^S^	**128 (2)** ^S^	**128 (2)** ^S^	**64 (4)** ^S^	**128 (2)** ^S^	**64 (2)** ^s^	256 (1)^I^	**75**
	MIC/4	256 (0.5)^a^	**64 (4)** ^S^	**128 (2)** ^S^	**128 (2)** ^S^	**128 (2)** ^S^	**128 (2)** ^S^	**64 (2)** ^s^	**128 (2)** ^S^	**87.5**

CLX	0	256	1024	> 1024	> 1024	1024	512	> 1024	> 1024	
	MIC/2	512 (0.5)^a^	> 1024 (> 1)^a^	**1024 (1)** ^S^	> 1024 (≥ 1)^I^	**512 (2)** ^S^	**128 (4)** ^S^	> 1024 (≥ 1)^I^	> 1024 (≥ 1)^I^	37.5
	MIC/4	512 (0.5)^a^	1024 (1)^I^	**1024 (1)** ^S^	> 1024 (≥ 1)^I^	**128 (8)** ^S^	> 1024 (> 0.5)^a^	> 1024 (≥ 1)^I^	**1024 (1)** ^S^	37.5

KAN	0	16	16	< 2	16	4	64	8	64	
	MIC/2	128 (0.125)^a^	128 (0.125)^a^	< 2 (≤ 1)^I^	**< 2 (> 8)** ^S^	**< 2 (> 2)** ^S^	**16 (4)** ^S^	16 (0.5)^a^	**16 (4)** ^S^	50
	MIC/4	128 (0.125)^a^	64 (0.25)^a^	< 2 (≤ 1)^I^	**< 2 (> 8)** ^S^	**< 2 (> 2)** ^S^	256 (025)^a^	16 (0.5)^a^	**4 (16)** ^S^	37.5

GEN	0	32	16	16	16	256	32	128	256	
	MIC/2	64 (0.5)^a^	> 256 (> 0.062)^a^	**4 (4)** ^S^	**< 2 (> 8)** ^S^	**< 2 (128)** ^S^	**16 (2)** ^S^	256 (0.5)^a^	**4 (64)** ^S^	62.5
	MIC/4	256 (0.125)^a^	> 256 (0.062)^a^	**4 (4)** ^S^	**< 2 (> 8)** ^S^	**< 2 (128)** ^S^	256 (0.125)^a^	256 (0.5)^a^	**4 (64)** ^S^	50

DOX	0	< 1	4	16	16	4	8	64	32	
	MIC/2	< 1 (≤ 1)^I^	8 (0.5)^a^	16 (1)^I^	16 (1)^I^	4 (1)^I^	**2 (4)** ^S^	**32 (2)** ^S^	**16 (2)** ^S^	37.5
	MIC/4	4 (0.25)^a^	4 (1)^I^	16 (1)^I^	16 (1)^I^	4 (1)^I^	**2 (4)** ^S^	**32 (2)** ^S^	**16 (2)** ^S^	37.5

CHL	0	2	32	16	256	64	32	256	64	
	MIC/2	2 (1)^I^	32 (1)^I^	16 (1)^I^	256 (1)^I^	64 (1)^I^	32 (1)^I^	256 (1)^I^	64 (1)^I^	0
	MIC/4	2 (1)^I^	**16 (2)** ^S^	16 (1)^I^	256 (1)^I^	64 (1)^I^	**16 (2)** ^S^	256 (1)^I^	64 (1)^I^	25

NOR	0	4	8	32	32	8	8	64	32	
	MIC/2	64 (0.062)^a^	32 (0.25)^a^	**16 (2)** ^S^	32 (1)^I^	64 (0.125)^a^	8 (1)^I^	**32 (2)** ^S^	32 (1)^I^	25
	MIC 4	64 (0.062)^a^	32 (0.25)^a^	32 (1)^I^	32 (1)^I^	32 (0.25)^a^	8 (1)^I^	**32 (2)** ^S^	32 (1)^I^	12.5

CIP	0	< 0.5	1	8	8	< 0.5	1	64	8	
	MIC/2	2 (0.25)^a^	8 (0.125)^a^	**1 (8)** ^S^	8 (1)^I^	8 (0.062)^a^	4 (0.25)^a^	**32 (2)** ^S^	**4 (2)** ^S^	37.5
	MIC/4	8 (0.062)^a^	4 (0.25)^a^	**2 (4)** ^S^	8 (1)^I^	2 (0.25)^a^	4 (0.25)^a^	**32 (2)** ^S^	**4 (2)** ^S^	37.5

ERY: erythromycin; CLX: cloxacillin; KAN: kanamycin; GEN: gentamycin; DOX: doxyciclin; CHL: chloramphenicol; NOR: norfloxacin; CIP: ciprofloxacin; bacterial strains: *Escherichia coli* (AG102, ATCC8739); *Enterobacter aerogenes* (ATCC13048, CM64); *Klebsiella pneumoniae* (KP55, KP63); *P. aeruginosa *PA124; *Providencia stuartii* NEA16; PBS: percentage of bacteria strain on which synergism has been observed. (): ameliorating factor of the antibiotics after association with compound **7**; S: synergy; I: indifference; a: antagonist; values in bold: case of synergy [19, 20].

**Table 6:** Antibiotic resistance modulatory activity of 3-*O*-[*β*-galactopyranosyl-(1→4)-*β*-_D_-galactopyranosyl]-oleanolic acid (**8**) against MDR Gram-negative bacteria.

Antibiotics	Extract concentration	Bacteria, MIC (*μ*g/mL) and modulating factors of compound **8** (in bracket)								
		ATCC8739	AG102	ATCC13048	CM64	KP55	KP63	PA124	NEA16	PBS (%)
ERY	0	128	256	256	256	256	256	256	256	
	MIC/2	256 (0.5)^a^	**128 (2)** ^S^	256 (1)^I^	**128 (2)** ^S^	**128 (2)** ^S^	**64 (4)** ^**S**^	**64 (4)** ^s^	**128 (2)** ^S^	**75**
	MIC/4	128 (1)^I^	**128 (2)** ^S^	**128 (2)** ^S^	256 (1)^I^	256 (1)^I^	**64 (4)** ^**S**^	**128 (2)** ^S^	256 (1)^I^	50

CLX	0	256	1024	> 1024	> 1024	1024	512	> 1024	> 1024	
	MIC/2	1024 (0.25)^a^	> 1024 (> 1)^a^	> 1024 (≥ 1)^I^	> 1024 (≥ 1)^I^	1024 (1)^I^	1024 (0.5)^a^	> 1024 (≥ 1)^I^	> 1024 (≥ 1)^I^	0
	MIC/4	> 1024(>0.25)^a^	> 1024 (> 1)^a^	> 1024 (≥ 1)^I^	> 1024 ≥ 1)^I^	1024 (1)^I^	> 1024 (> 0.5)^a^	> 1024 (≥ 1)^I^	> 1024 (≥ 1)^I^	0

KAN	0	16	16	< 2	16	4	64	8	64	
	MIC/2	**4 (4)** ^S^	64 (0.25)^a^	128 (0.015)^a^	128 (0.125)^a^	**< 2 (2)** ^S^	256 (0.25)^a^	8 (1)^I^	128 (0.5)^a^	25
	MIC/4	128 (0.125)^a^	32 (0.25)^a^	128 (0.015)^a^	128 (0.125)^a^	**< 2 (2)** ^S^	256 (0.25)^a^	8 (1)^I^	128 (0.5)^a^	12.5

GEN	0	32	16	16	16	256	32	128	256	
	MIC/2	256 (0.125)^a^	64 (0.25)^a^	128 (0.125)^a^	**8 (2)** ^S^	**64 (4)** ^S^	256 (0.125)^a^	**64 (2)** ^s^	256 (1)^I^	37.5
	MIC/4	256 (0.125)^a^	> 256 (> 0.062)^a^	128 (0.125)^a^	128 (0.125)^a^	**128(2)** ^S^	> 256 (>0.125)^a^	128 (1)^I^	**128 (2)** ^S^	25

DOX	0	< 1	4	16	16	4	8	64	32	
	MIC/2	< 1 (≤ 1)^I^	**2 (2)** ^S^	16 (1)^I^	32 (0.5)a	**< 1 (> 4)** ^S^	**2 (4)** ^**S**^	**32 (2)** ^s^	**16 (2)** ^S^	62.5
	MIC/4	< 1 (≤ 1) ^I^	4 (1)^I^	32 (0.5)a	32 (0.5)a	**< 1 (> 4)** ^S^	**2 (4)** ^**S**^	**32 (2)** ^s^	32 (1)^I^	37.5

CHL	0	2	32	16	256	64	32	256	64	
	MIC/2	2 (1)^I^	**16 (2)** ^S^	16 (1)^I^	256 (1)^I^	**32 (2)** ^S^	32 (1)^I^	256 (1)^I^	64 (1)^I^	25
	MIC/4	**1 (2)** ^S^	32(1)^I^	16 (1)^I^	256 (1)^I^	**32 (2)** ^S^	**16 (2)** ^**S**^	256 (1)^I^	64 (1)^I^	37.5

NOR	0	4	8	32	32	8	8	64	32	
	MIC/2	**2 (2)** ^S^	8 (1)^I^	> 128 (> 0.25)^a^	64 (0.5)^a^	**4 (2)** ^S^	8 (1)^I^	**32 (2)** ^S^	128 (0.125)^a^	37.5
	MIC/4	**< 1 (> 4)** ^S^	**2 (4)** ^S^	128 (0.25)^a^	64 (0.5)^a^	**4 (2)** ^S^	**4 (2)** ^**S**^	**32 (2)** ^S^	128 (0.125)^a^	62.5

CIP	0	< 0.5	1	8	8	< 0.5	1	64	8	
	MIC/2	< 0.5 (≤ 1)^I^	**< 0.5 (> 2)** ^S^	64 (0.125)^a^	64 (0.125)^a^	< 0.5 (≤ 1)^I^	4 (0.25)^a^	64 (1)^I^	64 (0.125)^a^	12.5
	MIC/4	2 (0.25)^a^	**< 0.5 (> 2)** ^S^	64 (0.125)^a^	64 (0.125)^a^	1 (2)^a^	2 (0.5)^a^	64 (1)^I^	64 (0.125)^a^	12.5

ERY: erythromycin; CLX: cloxacillin; KAN: kanamycin; GEN: gentamycin; DOX: doxyciclin; CHL: chloramphenicol; NOR: norfloxacin; CIP: ciprofloxacin; bacterial strains: *Escherichia coli* (AG102, ATCC8739); *Enterobacter aerogenes* (ATCC13048, CM64); *Klebsiella pneumonia* (KP55, KP63); *P. aeruginosa *PA124; *Providencia stuartii* NEA16; PBS: percentage of bacteria strain on which synergism has been observed. (): ameliorating factor of the antibiotics after association with compound **8**; S: synergy; I: indifference; a: antagonist; values in bold: case of synergy [19, 20].

## Data Availability

All data generated or analyzed during this study are included in this published article.

## Abbreviations

**1**: Stigmasterol
**2**: *β*-amyrin
**3**: Stigmasterol-3-*O*-*β*-glucopyranosyl
**4**: 3-O-methyl-D-chiro-inositol
**5**: Epicatechin
**6**: Quercetin-3-*O*-glucoside
**7**: 3-*O*-[*β*-_D_-xylopyranosyl-(1→4)-*β*-_D_-galactopyranosyl]-oleanolic acid
**8**: 3-*O*-[*β*-galactopyranosyl-(1→4)-*β*-_D_-galactopyranosyl]-oleanolic acid
AcOEt: Ethyl acetate
ATCC: American Type Culture Collection
APB: * Acacia polyacantha* bark extract
APL: * Acacia polyacantha* leaf extract
APLa-d: Fractions of* Acacia polyacantha* leaf extract
CC: Column chromatography
CIP: Ciprofloxacin
CHCl_3_: Chloroform
CHL: Chloramphenicol
CLX: Cloxacillin
DMSO: Dimethyl sulfoxide
DOX: Doxyciclin
EPI: Efflux pump inhibitor
*E. aerogenes*: * Enterobacter aerogenes*
*E. cloacae*: * Enterobacter cloacae*
*E. coli*: * Escherichia coli*
ERY: Erythromycin
GEN: Gentamycin
INT: * p*-iodonitrotetrazolium chloride
KAN: Kanamycin
*K. pneumoniae*: * Klebsiella pneumoniae*
MBC: Minimal bactericidal concentration
MDR: Multidrug resistant
MeOH: Methanol
MHB: Mueller Hinton Broth
MIC: Minimal inhibitory concentration
m.p.: Melting point
NMR: Nuclear magnetic resonance
NOR: Norfloxacin
PAßN: Phenylalanine-arginine-*β*-naphthylamide
*P. aeruginosa*: * Pseudomonas aeruginosa*
*P. stuartii*: * Providencia stuartii*
RA: Reference antibiotic.

## Supplementary Materials

Supplementary Materials
* Supplementary file.docx*.* S1:* RMN ^1^H, 13C and major chemical shifts of studied compounds;* S2:* bacterial strains and features;* S3:* preliminary evaluation of antibiotic resistance modulatory activity of selected samples at subinhibitory concentrations against* Pseudomonas aeruginosa *PA124 [1100 KB].
