# Supplementary material for: Antibacterial and Antibiotic Modifying Potential of Crude Extracts, Fractions, and Compounds from Acacia polyacantha Willd. against MDR Gram-Negative Bacteria
Source: Evid Based Complement Alternat Med. 2019 Mar 7;2019:7507549. doi: 10.1155/2019/7507549 (PMC6431460; doi:10.1155/2019/7507549)
Supplement: Supplementary Materials — Supplementary file.docx. S1: RMN 1H, 13C and major chemical shifts of studied compounds; S2: bacterial strains and features; S3: preliminary evaluation of antibiotic resistance modulatory activity of selected samples at subinhibitory concentrations against Pseudomonas aeruginosa PA124 [1100 KB]. [file 7507549.f1.docx]

**Antibacterial and antibiotic modifying potential of crude extracts, fractions and compounds from *Acacia polyacantha* Willd. against MDR Gram-negative bacteria**

Flora T. Mambe^1,2,3^, Jean Na-Iya^4^, Ghislain W. Fotso^4^, Fred Ashu^1,2^, Bathélémy Ngameni^5^, Bonaventure T. Ngadjui^4^, Veronique P. Beng^2^, Victor Kuete^1*^.

*^1^Department of Biochemistry, Faculty of Science, University of Dschang, Dschang, Cameroon;*

*^2^Department of Biochemistry, Faculty of Science, University of Yaoundé I, Yaoundé, Cameroon;*

*^3^Ministry of Scientific Research and Innovation;*

*^4^Department of Organic Chemistry, Faculty of Science, University of Yaoundé I, Yaoundé, Cameroon;*

*^5^Department of Pharmacognosy and Pharmaceutical Chemistry, Faculty of Medicine and Biomedical Science, University of Yaoundé I, Yaoundé, Cameroon.*

*****Corresponding author:**

*Tel : (237) 77 35 59 27; P.O. Box 67 Dschang, Cameroon; E-mail:* [*kuetevictor@yahoo.fr*](mailto:kuetevictor@yahoo.fr) *(Prof. Victor Kuete)*

**E-mails:**

*Flora T. Mambe:* [*latameghe07@yahoo.fr*](mailto:latameghe07@yahoo.fr)

*Jean Na-Iya:* [*jeannaiya2@gmail.com*](mailto:jeannaiya2@gmail.com)

*Ghislain W. Fotso:* [*ghis152001@yahoo.fr*](mailto:ghis152001@yahoo.fr)

*Fred Ashu:* [*ashufred50@yahoo.com*](mailto:ashufred50@yahoo.com)

*Bathelemy Ngameni:* [*bath_ngameni@yahoo.fr*](mailto:bath_ngameni@yahoo.fr)

*Bonaventure T. Ngadjui:* [*ngadjuibt@yahoo.fr*](mailto:ngadjuibt@yahoo.fr)

*Veronique P. Beng:* [*v.penlap@yahoo.fr*](mailto:v.penlap@yahoo.fr)

*Victor Kuete:* [*kuetevictor@yahoo.fr*](mailto:kuetevictor@yahoo.fr%20%20)

**S1.** RMN ^1^H, 13C and major chemical shifts of studied compounds

**Compound 1 (stigmastérol)**: RMN ^13^C (CDCl_3_, 150 MHz): 12,0 (C-_29_) ; 12,2 (C-_24_) ; 18,9 (C-_28_) ; 19,4 (C-_27_) ; 21,1 (C-_26_) ; 21,4 (C-_11_) ; 22,7 (C-_22_) ; 24,4 (C-_15_) ; 25,4 (C-_23_) ; 29,4 (C-_16_) ; 29,7 (C-_25_) ; 31,7 (C-_7_ et C-_8_) ; 31,9 (C-_2_) ; 36,5 (C-_10_) ; 37,2 (C-_1_) ; 39,7 (C-_12_) ; 40,6 (C-_18_) ; 42,2 (C-_13_) ; 42,3 (C-_4_) ; 50,2 (C-_22_) ; 51,2 (C-_9_) ; 55,9 (C-_17_) ; 56,8 (C-_14_) ; 71,8 (C-_3_) ; 121,7 (C-_6_) ; 129,3 (C-_21_) ; 138,3 (C-_20_) ; 140,7 (C-_5_) (Chaturvedula and Prakash, 2012).

RMN ^13^C (CDCl_3_, 150 MHz) of compound **1**

RMN ^1^H (CDCl_3_, 600 MHz) of compound **1**

**Compound 2 (β-amyrin):** RMN ^13^C (CDCl_3_, 150 MHz): 15,5 (C-25) ; 15,6 (C-24) ; 16,3 (C-26) ; 18,6 (C-6) ; 23,6 (C-11) ; 23,7 (C-30) ; 25,9 (C-27) ; 26,1(C-16) ; 26,2 (C-15) ; 26,6 (C-15) ; 27,2 (C-27) ; 28,0 (C-23) ; 28,4 (C-28) ; 31,0 (C-20) ; 32,5 (C-7) ; 32,6 (C-17) ; 33,8 (C-29) ; 34,7 (C-21) ; 36,9 (C-10) ; 37,1 (C-22) ; 38,6 (C-4) ; 38,8 (C-1) ; 39,8 (C-8) ; 41,7 (C-14) ; 46,8 (C-19) ; 47,2 (C-18) ; 47,6 (C-9) ; 55,2 (C-5) ; 79,0 (C-3) ; 121,7 (C-12) ; 145,2 (C-13) (Mahato and Kundu, 1994).

RMN ^13^C (CDCl_3_, 150 MHz) of compound **2**

RMN ^1^H (CDCl_3_, 600 MHz) of compound **2**

**Compound 3 (stigmasterol-3-*O-β*-glucopyranosyl)**

RMN ^13^C (C_5_D_5_N, 150 MHz) of compound **3**

RMN ^1^H (C_5_D_5_N, 600 MHz) of compound **3**

**Compound 4 (3-*O*-méthyl-D-Chiro-inositol):** RMN ^13^C (D_2_O, 150 MHz):59,6 (MeO-) ; 69,7 (C-_4_) ; 70,5 (C-_2_) ; 71,4 (C-_6_) ; 71,6 (C-_1_) ; 72,0 (C-_5_) ; 82,7 (C-_3_) (Sharma et al., 2016).

RMN ^1^H (D_2_O, 150 MHz) of compound **4**

RMN ^1^H (D_2_O, 600 MHz) of compound **4**

**Compound 5 (Epicatechin):** RMN^13^C (DMSO-*d_6_*, 150 MHz): 28,6(C-_4_) ; 65,3(C-_3_) ; 78,5(C-_2_) ; 94,5(C-_8_) ; 95,5 (C-_6_) ; 98,9 (C-_4a_) ; 115,2 (C-_2’_) ; 115,3 (C-_5’_) ; 118,4 (C-_6’_) ; 131,0 (C-_1’_) ; 144,8 (C-_4’_) ; 144,9 (C-_3’_) ; 156,1 (C-_8a_) ; 156,6 (C-_5_) ; 156,9 (C-_7_) (Adnyana et al., 2000).

RMN ^1^H (DMSO-*d_6_*, 150 MHz) of compound **5**

RMN ^1^H (DMSO-*d_6_*, 600 MHz) of compound **5**

**Compound 6 (quercetin-3-*O*-glucoside):** RMN ^13^C (DMSO-*d_6_*, 150 MHz): 60,6 (C-_6”_) ; 68,5 (C-_4”_); 71,5 (C-_2”_); 73,6 (C-_3”_); 76,3 (C-_5”_); 93,4 (C-_8_); 99,1 (C-_6_) ; 102,2 (C-_1”_);104,2 (C-_4a_) ; 115,6 (C-_2’_) ; 116,3 (C-_5’_); 121,5(C-_1’_) ; 122,4 (C-_6’_) ; 133,82 (C-_3_); 145,3 (C-_3’_) ; 148,9 (C-_4’_) ; 156,6 (C-_8a_) ; 156,7 (C-_2_) ; 161,6 (C-_5_) ; 164,7 (C-_7_) ;177,7 (C-_4_) (Xu et al., 2012).

RMN ^13^C (DMSO-*d_6_*, 150 MHz) of compound **6**

RMN ^1^H (DMSO-*d_6_*, 600 MHz) of compound **6**

**Compound 7 (3-*O-*[*β*-_D_-xylopyranosyl-(1→4)-*β*-_D_-galactopyranosyl]-oleanolic acid):** RMN ^13^C (CD_3_OD, 150 MHz): 14,5 (C-_25_) ; 15,6 (C-_24_) ; 16,3 (C-_26_) ; 17,9 (C-_6_) ; 22,6 (C-_30_) ; 22,7 (C-_16_) ; 23,1 (C-_11_) ; 25,0 (C-_27_) ; 25,6 (C-_2_) ; 27,1 (C-_23_) ; 27,4 (C-_15_) ; 30,2 (C-_20_) ; 32,2 (C-_29_) ; 32,4 (C-_22_) ; 32,6 (C-_7_) ; 33,5 (C-_21_) ; 36,5 (C-_10_) ; 38,4 (C-_1_) ; 38,7 (C-_4_) ; 39,2 (C-_18_) ; 41,3 (C-_8_) ; 41,5 (C-_14_) ; 45,8 (C-_19_) ; 46,2 (C-_17_) ; 47,6 (C-_9_) ; 55,6 (C-_5_) ; 60,6 (C-_6’_) ; 65,7 (C-_5’’_) ; 69,5 (C-_4’’_) ; 73,5 (C-_2’’_) ; 74,0 (C-_2’_) ; 74,6 (C-_5’_) ; 74,9 (C-_3’_) ; 76,4 (C-_3’’_) ;79,5 (C-_4’_) ; 89,5 (C-_3_) ; 104,0 (C-_1’’_) ; 105,1 (C-_1’_) ; 122,2 (C-_12_) ; 143,8 (C-_13_) ; 180,5 (C-_28_) (Fotso et al., 2018).

RMN ^13^C (CD_3_OD, 600 MHz) of compound **7**

RMN ^1^H (CD_3_OD, 600 MHz) of compound **7**

**Compound 8 (3-*O*-[*β*-galactopyranosyl-(1→4)-*β*-_D_-galactopyranosyl]-oleanolic acid:** RMN ^13^C (CD_3_OD, 150 MHz):14,5 (C-_25_) ; 15,5 (C-_24_) ; 16,3 (C-_26_) ; 18,0 (C-_6_) ; 22,7 (C-_30_) ; 23,1 (C-_16_) ; 25,0 (C-_11_) ; 25,7 (C-_27_) ; 27,1 (C-_2_ et C-_23_) ; 27,4 (C-_15_) ; 32,2 (C-_20_) ; 32,4 (C-_7_) ; 32,6 (C-_22_et C-_29_) ; 33,5 (C-_21_) ; 36,5 (C-_10_) ; 38,4 (C-_4_) ; 39,2 (C-_1_) ; 39,0 (C-_8_) ; 41,4 (C-_18_) ; 41,5 (C-_14_) ; 45,9 (C-_17_) ; 45,7 (C-_9_) ; 46,3 (C-_19_) ; 55,6 (C-_5_) ; 61,7 (C-_6’_) ; 70,1 (C-_4’’_) ; 70,4 (C-_2’’_) ;74,9 (C-_3’_) ;76,2 (C-_3’’_) ;76,5 (C-_2’_) ; 77,0 (C-_5’_) ; 77,1 (C-_5’’_) ; 79,7 (C-_4’_) ; 90,1 (C-_3_) ; 103,1 (C-_1’_) ; 104,0 (C-_1’’_) ; 122,2 (C-_12_) ; 143,8 (C-_13_) ; 180,7 (C-_28_) (Chaturvedula and Prakash, 2012).


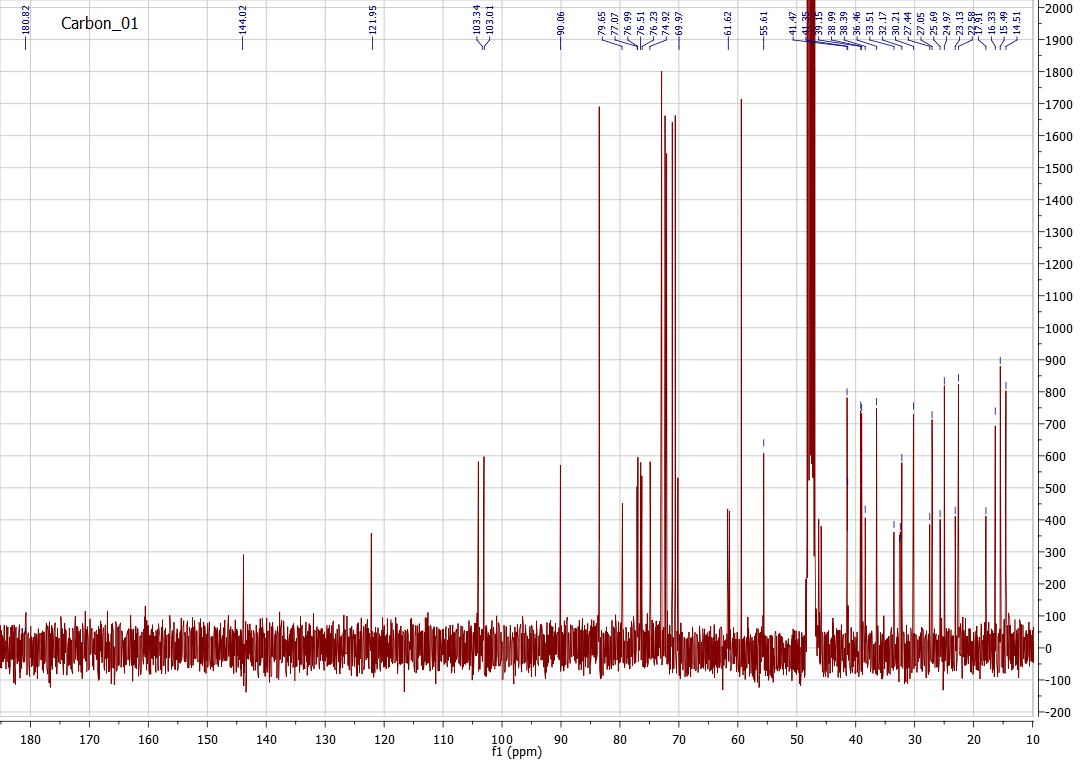


RMN ^13^C (CD_3_OD, 600 MHz) of compound **8**


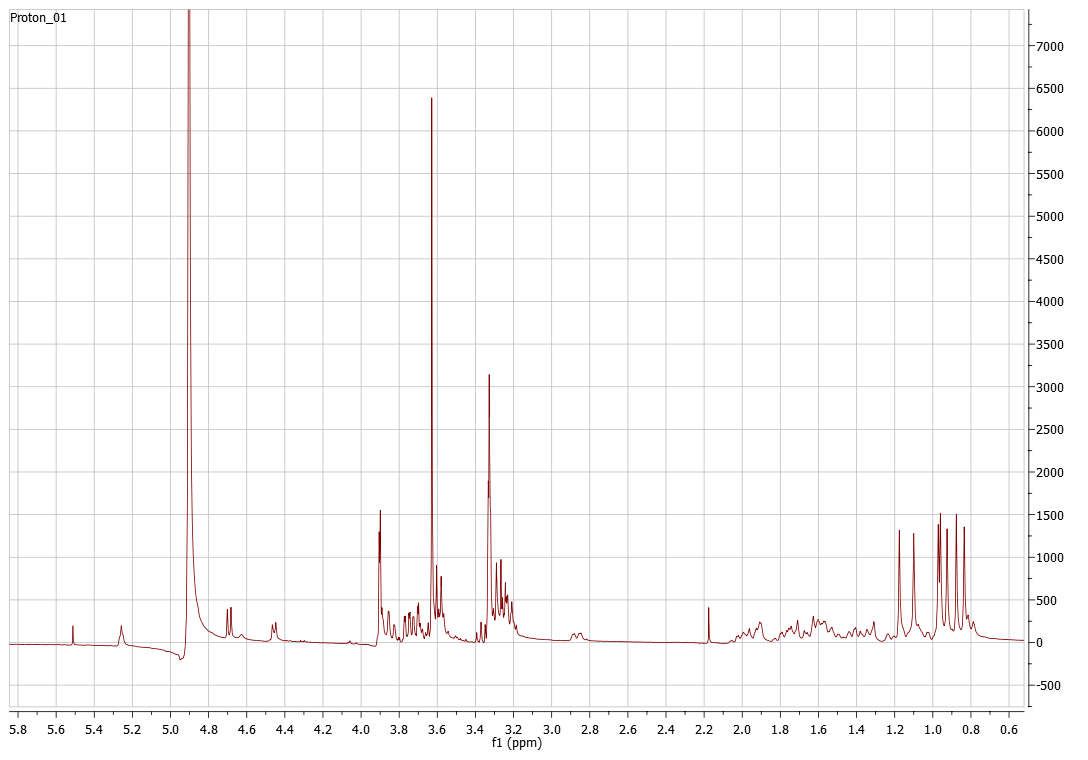


RMN ^1^H (CCl_3_OD, 600 MHz) of compound **8**

**S2.** Bacterial strains and features

| **Espèces** | **Souches** | **Caractéristiques** | | **Références** |
| --- | --- | --- | --- | --- |
| ***Escherichia***  ***coli*** | ATTC 8739 | Reference strain | |  |
|  | AG 100 | Wild-type *E. coli* K-12 expressing *Acr AB* efflux pumps | | (Viveiros et al., 2005) |
|  | AG 100ATet | ΔacrAB mutant AG 100A Tet^r^ | | (Viveiros et al., 2005) |
|  | AG 102 | AG 100 expressing *Acr AB* pumps | | (Elkins and Mullis, 2007) |
|  |  |  |  |  |
|  |  |  |  |  |
| ***Enterobacter aerogenes*** | ATCC 13048 | Reference strain | |  |
|  |  |  |  |  |
|  | EA 27 | Clinical MDR isolate exhibiting  energy-dependent norfloxacin and  chloramphenicol efflux with KAN^R^ and  AMP^R^ and NAL^R^ and STR^R^ and TET^R^ | | (Mallea et al., 1998; Mallea et al., 2003) |
|  | EA 289 | KAN sensitive derivative of EA27 | | (Ghisalberti et al., 2005) |
|  |  |  |  |  |
|  |  |  |  |  |
| ***Klebsiella pneumoniae*** | ATCC11296 | Reference strain | |  |
|  |  |  |  |  |
|  | Kp 55 | Clinical MDR isolate, TET^R^, AMP^R^, ATM^R^, and CEF^R^ | | (Chevalier et al., 2000) |
|  | Kp 63 | Clinical MDR isolate, TET^R^, CHL^R^, AMP^R^, and ATM^R^ | | (Chevalier et al., 2000) |
| ***Providencia stuartii*** | NEA 16 | Clinical MDR isolate of *Providencia stuartii* expressing *AcrAB-TolC* | | (Tran et al., 2010) |
|  |  | |  |  |
| ***Pseudomonas aeruginosa*** | PA 01 | Reference strain | |  |
|  | PA 124 | MDR clinical isolate *expressing MexAB-OprMpump* | | (Lorenzi et al., 2009) |

AMP^r^, ATM^r^, CEF^r^, CHL^r^, KAN^r^, NAL^r^, NOR^r^, STR^r^ et TET^r^, resistance to:ampicilline, aztreonam, céfépime, chloramphenicol, kanamycine, acidenalidixique, norfloxine, streptomycine and tétracycline respectively ;*AcrAB-TolC, MexAB-OprM :* pumps efflux.

**S3.** Preliminary evaluation of antibiotic-resistance modulatory activity of selected samples at sub-inhibitory concentrations against *Pseudomonnas aeruginosa PA124*

| **Plant extract** | | **Extract concentration** | **MIC of antibiotic (μg/mL) alone and in combination with extracts and fold increase of activity (in bracket)** | | | | | | | |
| --- | --- | --- | --- | --- | --- | --- | --- | --- | --- | --- |
|  |  | | **KAN NFX CIP CLX DOX ERY CHL GEN** | | | | | | | |
|  | | 0 | 8 | 64 | 64 | > 1024 | 64 | 256 | 256 | 128 |
| **APL** | | MIC /2 | 8 (1)^I^ | **32 (2)^S^** | **16 (4)**^S^ | > 1024 (≥ 1)^I^ | **32 (2)^S^** | > 256 (> 1)^a^ | 256 (1)^I^ | **64 (2)^s^** |
|  |  | MIC /4 | 8 (1)^I^ | **32 (2)^S^** | **16 (4)^S^** | > 1024 (≥ 1)^I^ | 64 (1) ^I^ | 256 (1)^I^ | 256 (1)^I^ | **64 (2)^s^** |
|  |  | MIC /8 | 8 (1)^I^ | 64 (1)^I^ | 64 (1) | > 1024 (≥ 1)^I^ | 64 (1) ^I^ | > 256 (> 1)^a^ | 256 (1)^I^ | 128 (1)^I^ |
|  |  | MIC /16 | 8 (1)^I^ | 64 (1)^I^ | 64 (1) ^I^ | > 1024 (≥ 1)^I^ | 64 (1) ^I^ | 256 (1) ^I^ | 256 (1)^I^ | 128 (1)^I^ |
| **APB** | | MIC /2 | >256 (< 0.031)^a^ | > 128 (<0.5)^a^ | > 64 (< 1)^a^ | > 1024 (≥ 1)^I^ | **32 (2)^s^** | **64 (4)^s^** | 256 (1)^I^ | > 256 (> 1)^a^ |
|  |  | MIC /4 | 16 (0.5)^a^ | > 128 (<0.5)^a^ | 64 (1) ^I^ | > 1024 (≥ 1)^I^ | **32 (2)^s^** | **64 (4)^s^** | 256 (1)^I^ | > 256 (> 0.5)^a^ |
|  |  | MIC /8 | 8 (1)^I^ | > 128 <0.5)^a^ | 64 (1)^I^ | > 1024 (≥ 1)^I^ | 64 (1)^I^ | > 256 (> 1)^a^ | 256 (1)^I^ | > 256 (> 0.5)^a^ |
|  |  | MIC /16 | 8 (1)^I^ | > 128 (<0.5)^a^ | 64 (1) ^I^ | > 1024 (≥ 1)^I^ | 64 (1)^I^ | > 256 (> 1)^a^ | 256 (1) | 128 (1) ^I^ |
| **Compound 1** | | MIC /2 | 8 (1)^I^ | 64 (1) ^I^ | > 64 (< 1)^a^ | **1024 (1)^S^** | **32 (2)^s^** | 256 (1) ^I^ | 256 (1)^I^ | 128 (1) ^I^ |
|  |  | MIC /4 | 8 (1)^I^ | 64 (1) ^I^ | 64 (1) ^I^ | **1024 (1) ^S^** | **32 (2)^s^** | 256 (1) ^I^ | 256 (1)^I^ | 256 (0.5)^a^ |
|  |  | MIC /8 | 8 (1)^I I^ | 64 (0.5)^a^ | 64 (1) ^I^ | **1024 (1) ^S^** | 64 (1)^I^ | 256 (1)^I^ | 256 (1)^I^ | 128 (1)^I^ |
|  |  | MIC /16 | 8 (1)^I^ | 64 (0.5)^a^ | 64 (1) ^I^ | **1024 (1) ^S^** | 64 (1) ^I^ | > 256 (> 1)^a^ | 256 (1)^I^ | **64 (2)^s^** |
| **Compound 2** | | MIC /2 | 64 (0.25)^a^ | 64 (1) ^I^ | 64 (1) ^I^ | > 1024 (≥ 1)^I^ | 64 (1) ^I^ | 256 (1) ^I^ | 256 (1)^I^ | 128 (1)^I^ |
|  |  | MIC /4 | 8 (1)^I^ | 64 (1) ^I^ | 64 (1) ^I^ | > 1024 (≥ 1)^I^ | 64 (1) ^I^ | 256 (1) ^I^ | 256 (1)^I^ | 128 (1) ^I^ |
|  |  | MIC /8 | 8 (1)^I^ | 64 (1) ^I^ | 64 (1) ^I^ | > 1024 (≥ 1)^I^ | 64 (1) ^I^ | 256 (1) ^I^ | 256 (1)^I^ | 128 (1) ^I^ |
|  |  | MIC /16 | 8 (1)^I^ | 64 (1) ^I^ | 64 (1) ^I^ | > 1024 (≥ 1)^I^ | 64 (1) ^I^ | 256 (1) ^I^ | 256 (1)^I^ | 128 (1) ^I^ |
| **Compound 3** | | MIC /2 | 8 (1)^I^ | **32 (2)^S^** | **32 (2)^S^** | >1024 (≥ 1)^I^ | 64 (1)^I^ | 256 (1) ^I^ | 256 (1)^I^ | 128 (1) ^I^ |
|  |  | MIC /4 | 8 (1)^I^ | **32 (2)^S^** | **32 (2)^S^** | > 1024 (≥ 1)^I^ | 64 (1)^I^ | 256 (1) ^I^ | 256 (1)^I^ | 128 (1) ^I^ |
|  |  | MIC /8 | 8 (1)^I^ | **32 (2)^S^** | **32 (2)^S^** | > 1024 (≥ 1)^I^ | 64 (1)^I^ | 256 (1) ^I^ | 256 (1)^I^ | 128 (1) ^I^ |
|  |  | MIC /16 | 8 (1)^I^ | **32 (2)^S^** | **32 (2)^S^** | > 1024 (≥ 1)^I^ | 64 (1) ^I^ | 256 (1)^I^ | 256 (1)^I^ | 128 (1) ^I^ |

() : modulating factor ; a : antagoniste ; I : indifférence ; s : synergy ; MIC= > 1024µg/ml for the leaves and bark, > 256 µg/mL for compounds **1, 2** and **3**; CHL: chloramphenicol, CIP: ciprofloxacin; ERY: erythromycin, KAN: kanamycin, GEN: Gentamycin, NFX: norfloxacin; DOX: doxyciclin ; CLX : cloxacillin.

**S2.** Preliminary evaluation of antibiotic-resistance modulatory activity of selected samples at sub-inhibitory concentrations against *Pseudomonnas aeruginosa PA124 (continue and end)*

| **Plant extract** | **Extract concentration** | | **MIC of antibiotic (μg/mL) alone and in combination with extracts and fold increase of activity (in bracket)** | | | | | | | |
| --- | --- | --- | --- | --- | --- | --- | --- | --- | --- | --- |
|  | | | **KAN NFX CIP CLX DOX ERY CHL GEN** | | | | | | | |
|  | | 0 | 8 | 64 | 64 | > 1024 | 64 | 256 | 256 | 128 |
| **Compound 4** | | MIC /2 | 8 (1)^I^ | 128 (0.5)^a^ | **8 (4)^s^** | > 1024 (≥1)^I^ | 64 (1) ^I^ | **128 (2)^s^** | 256 (1)^I^ | 128 (1)^I^ |
|  |  | MIC /4 | 8 (1)^I^ | 128 (0.5)^a^ | **8 (4)^s^** | > 1024 (≥1)^I^ | 128 (0.5)^a^ | 256 (1) ^I^ | 256 (1)^I^ | 128 (1)^I^ |
|  |  | MIC /8 | 8 (1)^I^ | 64 (1)^I^ | 32 (1)^I^ | >1024 (≥ 1)^I^ | 64 (1)^I^ | 256 (1) ^I^ | 256 (1)^I^ | 128 (1)^I^ |
|  |  | MIC /16 | 8 (1)^I^ | 64 (1) ^I^ | 32 (1) ^I^ | > 1024 (≥1)^I^ | 64 (1) ^I^ | 256 (1)^I^ | 256 (1)^I^ | 128 (1)^I^ |
| **Compound 5** | | MIC /2 | 8 (1)^I^ | **32 (2)^s^** | 32 (1)^I^ | >1024 (≥ 1)^I^ | 64 (1)^I^ | 256 (1)^I^ | 256 (1)^I^ | 128 (1)^I^ |
|  |  | MIC /4 | 8 (1)^I^ | **32 (2)^s^** | 32 (1)^I^ | >1024 (≥ 1)^I^ | 64 (1)^I^ | 256 (1)^I^ | 256 (1)^I^ | 128 (1)^I^ |
|  |  | MIC /8 | 8 (1)^I^ | **32 (2)^s^** | 32 (1)^I^ | >1024 (≥ 1)^I^ | 64 (1)^I^ | 256 (1)^I^ | 256 (1)^I^ | 128 (1)^I^ |
|  |  | MIC /16 | 8 (1)^I^ | **32 (2)^s^** | 32 (1)^I^ | >1024 (≥ 1)^I^ | 64 (1)^I^ | 256 (1)^I^ | 256 (1)^I^ | 128 (1)^I^ |
| **Compound 6** | | MIC /2 | 128 (0.062)^a^ | 128 (0.5)^a^ | 32 (1) ^I^ | >1024 (≥ 1)^I^ | **32 (2)^s^** | > 256 (< 1)^a^ | 256 (1)^I^ | **64 (2)^s^** |
|  |  | MIC /4 | 128 (0.062)^a^ | 128 (0.5)^a^ | 32 (1) ^I^ | > 1024 (≥1)^I^ | **32 (2)^s^** | > 256 (< 1)^a^ | 256 (1)^I^ | 128 (1) ^I^ |
|  |  | MIC /8 | 128 (0.062)^a^ | 128 (0.5)^a^ | 32 (1) ^I^ | > 1024 (≥1)^I^ | 64 (1) ^I^ | > 256 (< 1)^a^ | 256 (1)^I^ | 256 (0.5)^a^ |
|  |  | MIC /16 | 128 (0.062)^a^ | 128 (0.5)^a^ | > 64 (> 0.5)^a^ | > 1024 (≥1)^I^ | 64 (1) ^I^ | >256 (<1)^a^ | 256 (1)^I^ | 128 (1) ^I^ |
| **Compound 7** | | MIC /2 | 16 (0.5)^a^ | **32 (2)^S^** | **32 (2)^S^** | >1024 (≥ 1)^I^ | **32 (2)^s^** | **64 (2)^s^** | 256 (1)^I^ | 256 (0.5)^a^ |
|  |  | MIC /4 | 16 (0.5)^a^ | **32 (2)^S^** | **32 (2)^S^** | >1024 (≥ 1)^I^ | **32 (2)^s^** | **64 (2)^s^** | 256 (1)^I^ | 256 (0.5)^a^ |
|  |  | MIC /8 | 16 (0.5)^a^ | 64 (1)^I^ | 64 (1)^I^ | >1024 (≥ 1)^I^ | 64 (1) ^I^ | 256 (1)^I^ | 256 (1)^I^ | 256 (0.5)^a^ |
|  |  | MIC /16 | 16 (0.5)^a^ | 64 (1)^I^ | 64 (1)^I I^ | >1024 (≥ 1)^I^ | 64 (1) ^I^ | 256 (1)^I^ | 256 (1)^I^ | 256 (0.5)^a^ |
| **Compound 8** | | MIC /2 | 8 (1)^I^ | **32 (2)^S^** | 64 (1)^I^ | >1024 (≥ 1)^I^ | **32 (2)^s^** | **64 (4)^s^** | 256 (1)^I^ | **64 (2)^s^** |
|  |  | MIC /4 | 8 (1)^I^ | **32 (2)^S^** | 64 (1)^I^ | > 1024 (≥1)^I^ | **32 (2)^s^** | **128 (2)^s^** | 256 (1)^I^ | 128 (1) ^I^ |
|  |  | MIC /8 | 8 (1)^I^ | 64 (1)^I^ | 64 (1)^I^ | > 1024 (≥1)^I^ | **32 (2)^s^** | **64 (4)^s^** | 256 (1)^I^ | 256 (0.5)^a^ |
|  |  | MIC /16 | 8 (1)^I^ | >128 (<0.5)^a^ | 64 (1) ^I^ | > 1024 (≥1)^I^ | **32 (2)^s^** | 256 (1)^I^ | 256 (1)^I^ | 256 (0.5)^a^ |

() : modulating factor ; a : antagoniste ; I : indifférence ; s : synergy ; MIC= > 256 µg/mL for compounds **4, 6** and **7**; MIC = 64µg/mL for **5**; MIC = 32µg/mL for **8**; CHL: chloramphenicol, CIP: ciprofloxacin; ERY: erythromycin, KAN: kanamycin, GEN: Gentamycin, NFX: norfloxacin; DOX: doxyciclin ; CLX : cloxacillin.

**References**

Adnyana, I.K., Tezuka, Y., Awale, S., Banskota, A.H., Tran, K.Q., Kadota, S., 2000. Quadranosides VI-XI, six new triterpene glucosides from the seeds of *Combretum quadrangulare*. Chem Pharm Bull (Tokyo) 48, 1114-1120.

Chaturvedula, V.S.P., Prakash, I., 2012. Isolation of stigmasterol and β-Sitosterol from the dichloromethane extract of *Rubus suavissimus.* International Current Pharmaceutical Journal 1, 239-242.

Chevalier, J., Pages, J.M., Eyraud, A., Mallea, M., 2000. Membrane permeability modifications are involved in antibiotic resistance in *Klebsiella pneumoniae*. Biochem Biophys Res Commun 274, 496-499.

Elkins, C.A., Mullis, L.B., 2007. Substrate competition studies using whole-cell accumulation assays with the major tripartite multidrug efflux pumps of *Escherichia coli*. Antimicrob Agents Chemother 51, 923-929.

Fotso, W.G., Na-Iya, J., Mbaveng, T.A., Ango Yves, P., Demirtas, I., Kuete, V., Samuel, Y., Ngameni, B., Efferth, T., Ngadjui, T.B., 2018. Polyacanthoside A, a new oleanane-type triterpenoid saponin with cytotoxic effects from the leaves of *Acacia polyacantha* (Fabaceae). Nat Prod Res, 1-6.

Ghisalberti, D., Masi, M., Pages, J.M., Chevalier, J., 2005. Chloramphenicol and expression of multidrug efflux pump in *Enterobacter aerogenes*. Biochem Biophys Res Commun 328, 1113-1118.

Lorenzi, V., Muselli, A., Bernardini, A.F., Berti, L., Pages, J.M., Amaral, L., Bolla, J.M., 2009. Geraniol restores antibiotic activities against multidrug-resistant isolates from gram-negative species. Antimicrob Agents Chemother 53, 2209-2211.

Mahato, S.B., Kundu, S.P., 1994. 13C NMR spectra of pentacyclic triterpenoids-A compilation and some salient features. Phytochemistry 37, 1517-1575.

Mallea, M., Chevalier, J., Bornet, C., Eyraud, A., Davin-Regli, A., Bollet, C., Pages, J.M., 1998. Porin alteration and active efflux: two in vivo drug resistance strategies used by *Enterobacter aerogenes*. Microbiology 144 ( Pt 11), 3003-3009.

Mallea, M., Mahamoud, A., Chevalier, J., Alibert-Franco, S., Brouant, P., Barbe, J., Pages, J.M., 2003. Alkylaminoquinolines inhibit the bacterial antibiotic efflux pump in multidrug-resistant clinical isolates. Biochem J 376, 801-805.

Sharma, N., Verma, M.K., Gupta, D.K., Satti, N.K., Khajuria, R.K., 2016. Isolation and quantification of D-pinitolin *Argyrolobium roseum* plant, by 1H-NMR. Journal of Saudi Chemical Society 20, 81-87.

Tran, Q.T., Mahendran, K.R., Hajjar, E., Ceccarelli, M., Davin-Regli, A., Winterhalter, M., Weingart, H., Pages, J.M., 2010. Implication of porins in beta-lactam resistance of *Providencia stuartii.* J Biol Chem 285, 32273-32281.

Viveiros, M., Jesus, A., Brito, M., Leandro, C., Martins, M., Ordway, D., Molnar, A.M., Molnar, J., Amaral, L., 2005. Inducement and reversal of tetracycline resistance in Escherichia coli K-12 and expression of proton gradient-dependent multidrug efflux pump genes. Antimicrob Agents Chemother 49, 3578-3582.

Xu, L.W., Chen, J., Qi, H.Y., Shi, Y.P., 2012. Phytochemicals and their biological activities of plants in *Tagetes* L. . Chinese Herbal Medicines 4, 103-117.
